# Diagnostic and therapeutic challenges of a “minimally invasive penetrating foreign body”: a case report

**DOI:** 10.3389/fsurg.2026.1796341

**Published:** 2026-05-13

**Authors:** Wenjin Zhang, Cheng Liu, Changsong Wu, Tingting Hao

**Affiliations:** Department of Otolaryngology–Head and Neck Surgery, Anhui No.2 Provincial People’s Hospital, Hefei, Anhui, People’s Republic of China

**Keywords:** case report, endoscopy, esophageal foreign bodies, surgical exploration, treatment

## Abstract

Missed diagnosis and treatment of esophageal foreign bodies (FBs) carry a risk of severe, life-threatening complications. This case report details the diagnostic and therapeutic management of a 58-year-old woman with a minimally invasive penetrating FB (MIPFB) following fish bone ingestion, who presented with odynophagia. Initial clinical evaluation and electronic nasopharyngolaryngoscopy were unremarkable. A cervical computed tomography scan on day 6 post-ingestion revealed a penetrating FB at the C6 vertebral level. Initial flexible esophagoscopy and a subsequent surgical exploration failed to localize the object. The FB, an approximately 2.5-cm-long fish bone embedded in the right posterior wall of the esophageal inlet, was ultimately and successfully retrieved via a repeated, meticulous endoscopic procedure employing targeted mucosal manipulation. This case highlights that MIPFB cases are prone to diagnostic delay and emphasizes that repeated, careful endoscopy is a superior and less invasive therapeutic strategy compared to surgical exploration in such complex scenarios.

## 1 Introduction

Esophageal foreign bodies represent emergencies in otolaryngology and gastroenterology. Fish bones are the most commonly ingested foreign bodies in adults, typically lodging in the oropharynx, hypopharynx, or esophagus [1]. Delayed management can cause perforation, hemorrhage, aortoesophageal fistula, tracheoesophageal fistula, mediastinal sepsis, and other related complications [2]. While endoscopy succeeds in >93% of cases [3–6], false negatives may still occur. We present a case of a minimally invasive penetrating foreign body (MIPFB) to elucidate diagnostic and therapeutic challenges. In this study, we propose the descriptive term “minimally invasive penetrating foreign body” to describe the condition observed in our case, where a foreign body has penetrated the wall of a hollow viscus but elicits only minimal symptoms and a local tissue reaction.

## 2 Case report

**Figure 1:**
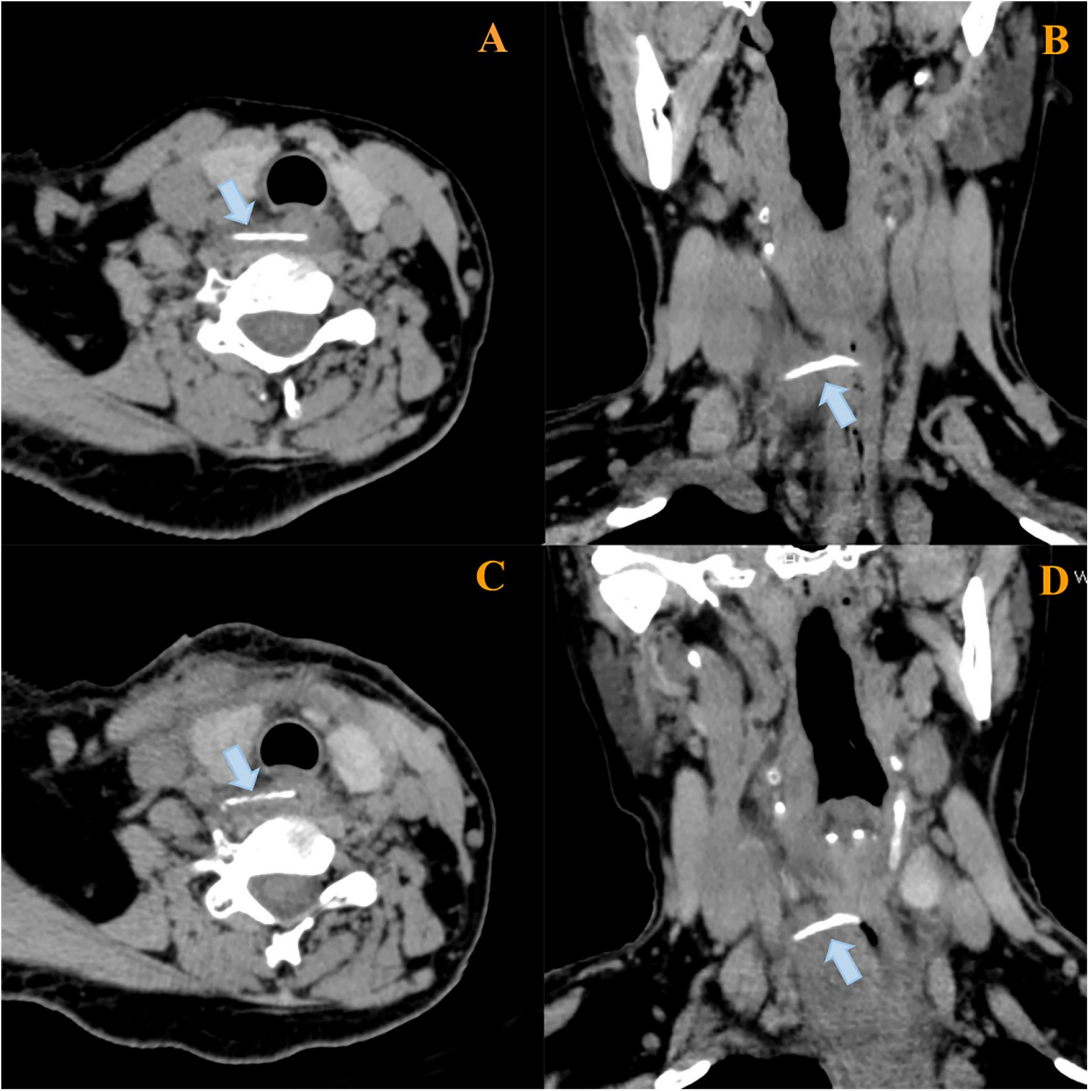
Preoperative and follow-up CT images were acquired without intravenous contrast. Axial and coronal multiplanar reformation (MPR) CT images obtained preoperatively **(A,B)** and at follow-up **(C,D)**. The preoperative images demonstrate a linear hyperdense foreign body penetrating the wall of the cervical esophageal inlet (at the C6 superior margin) into the right periesophageal space, surrounded by ill-defined fat stranding and patchy exudative densities. The distal end of the foreign body was approximately 3.5 mm from the right common carotid artery. The follow-up images confirm the foreign body’s persistent location. Compared to the initial study, there is increased periesophageal fat stranding and patchy exudation, with the distance to the right common carotid artery measuring approximately 3.2 mm.

**Figure 2:**
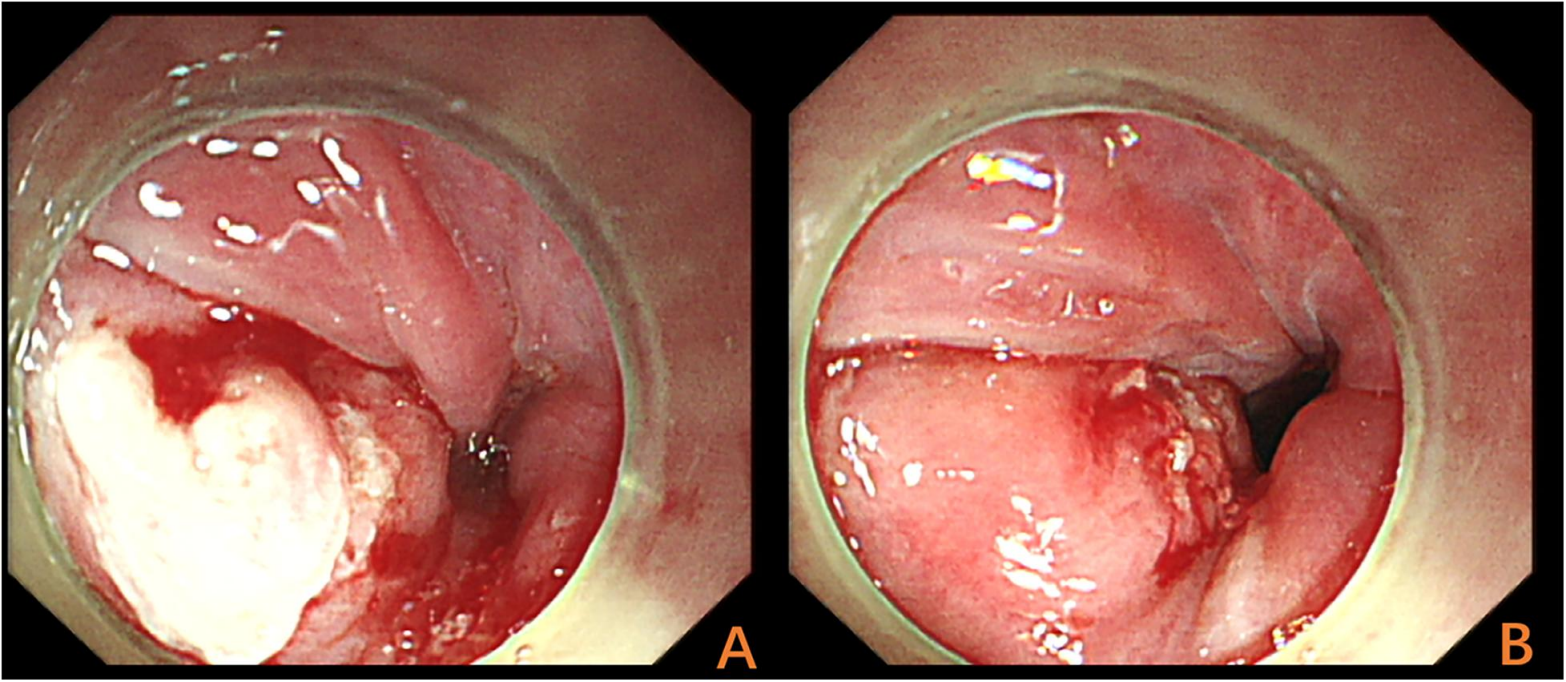
First flexible esophagoscopy inspection images. Initial Flexible esophagoscopy shows only mucosal edema along with a localized superficial ulcer at the esophageal inlet **(A)**; after debridement of the pseudomembrane, no foreign body was identified **(B)**.

A 58-year-old woman presented with a 6-day history of odynophagia following fish bone ingestion. Physical examination was unremarkable. The patient declined initial nasopharyngolaryngoscopy and self-administered antibiotics. Due to persistent yet atypical symptoms, a cervical computed tomography (CT) scan was obtained directly on the day of admission (1 April 2025), bypassing conventional radiography for its higher sensitivity. The CT scan revealed an approximately 2.5-cm-long linear foreign body at the C6 level, penetrating the esophageal wall with the tip embedded submucosally and the main body located extra-esophageally (Figures 1A,B). An emergency flexible esophagoscopy performed on the same day demonstrated only mucosal edema and a localized superficial ulcer at the esophageal inlet; no foreign body was directly identified (Figure 2).

**Figure 3:**
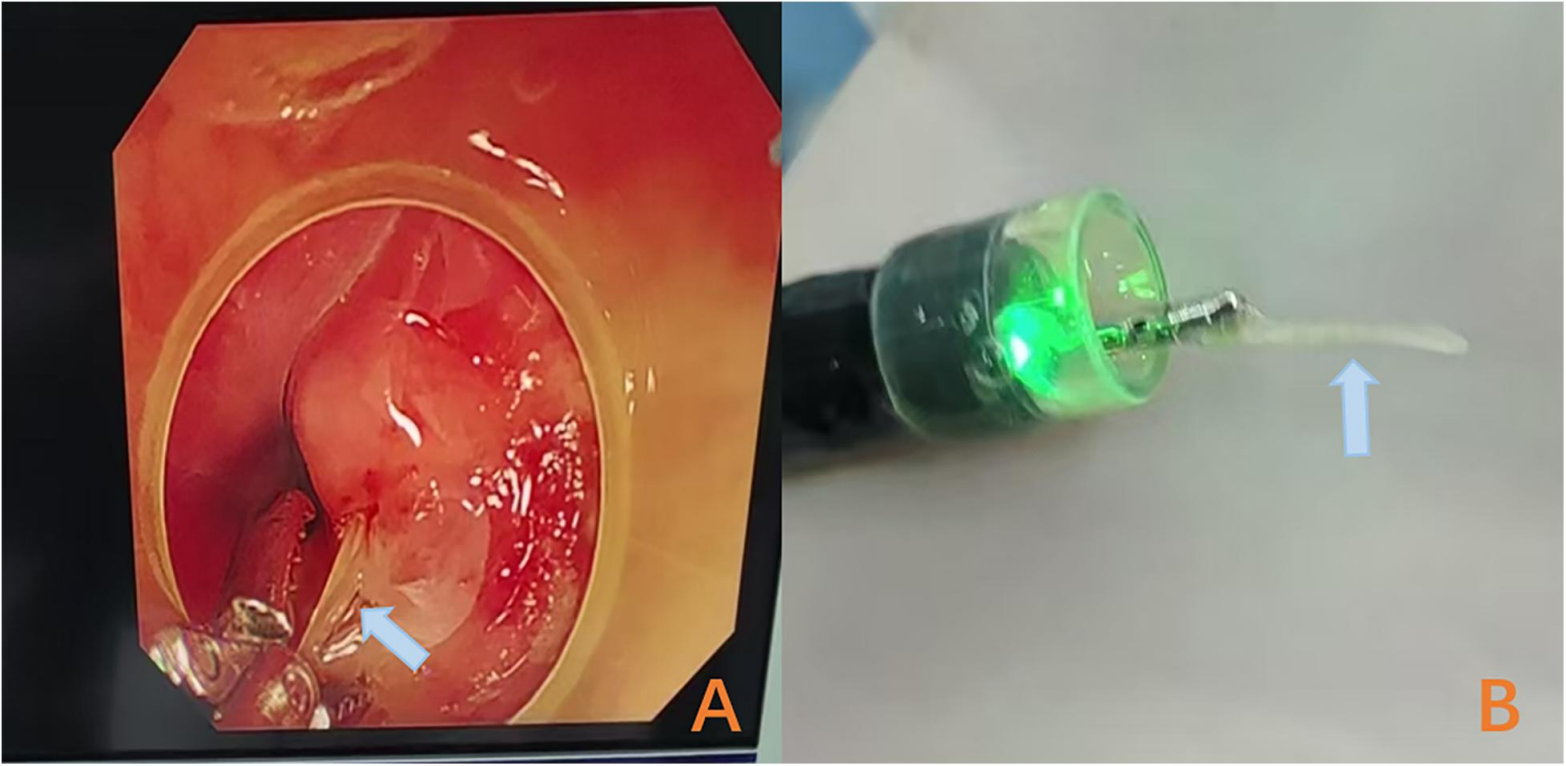
Second flexible esophagoscopy inspection images. Directed mucosal manipulation at the right posterior esophageal inlet successfully extracted a fish bone **(A,B)**.

Surgical exploration via a right cervical approach was performed under general anesthesia on the following day (2 April 2025) after multidisciplinary consultation. The procedure, which included esophageal mobilization, C-arm fluoroscopic guidance, and assistance from cardiothoracic and orthopedic surgeons, failed to locate the object. A repeat CT scan on postoperative day 5 confirmed its unchanged position (Figures 1C,D). Consequently, a second flexible endoscopic procedure was undertaken on 9 April 2025. During this procedure, focused attention on the right posterior esophageal inlet allowed for the successful extraction of the fish bone from the site of subtle edema via directed mucosal manipulation (Figure 3). The patient resumed oral intake within 24 h and was discharged uneventfully.

## 3 Discussion

In adults, foreign body ingestion is typically accidental, predominantly involving fish bones in Asian populations [7]. Patient symptoms, such as pain, are often non-specific [8]. Delayed management can lead to severe complications, including perforation, hemorrhage, aortoesophageal fistula, tracheoesophageal fistula, and mediastinal sepsis [2]. Particular attention is warranted for foreign bodies impacted in the proximal esophagus due to the risk of acute airway compromise, which can occur either from direct compression of the posterior tracheal wall causing respiratory distress, or from associated esophageal obstruction increasing the risk of aspiration [9]. Therefore, our standard approach for symptoms above the thyroid is initial laryngoscopy, followed by CT if negative. For objects below the pyriform sinus, we recommend flexible or rigid esophagoscopy. Surgical exploration is reserved for the <1% of cases with large, migrated, or complicated foreign bodies where endoscopy fails [10, 11].

Given the well-documented low sensitivity (25%–32%) of soft-tissue neck radiography and the potential for barium contrast to obscure the endoscopic field, cervical CT has become our routine first-line imaging modality due to its diagnostic accuracy (sensitivity 100%, specificity 97.8%) in confirming the presence of impacted bone [12, 13]. All examinations were performed on a high-resolution CT scanner (Philips Brilliance 64) without intravenous contrast, using the following parameters: helical pitch, 3 mm; tube current, 300 mA; tube voltage, 120 kV; matrix, 512×512; and a volume CT dose index (CTDIvol) of 19.57 mGy per scan. Axial data were reconstructed with a standardized sharp kernel, and thin-slice images (1 mm thickness and interval) were generated. Three-dimensional (3D) reformations were created as needed. This imaging protocol enables precise delineation of the foreign body’s spatial relationship to adjacent vital structures, thereby directly informing procedural planning and risk stratification.

Although CT was invaluable for diagnosis and preoperative planning, in this case, the submucosal embedding of the MIPFB at the C6 inlet posed significant challenges for therapeutic assessment. Hence, meticulous attention to indirect CT signs (e.g., subtle edema and soft tissue wrapping) is crucial. In addition, intraoperative C-arm fluoroscopy provided suboptimal visualization, likely hampered by sterile field constraints limiting optimal patient positioning.

This case deviates from the typical clinical course. Guidelines recommend endoscopy within 24 h for sharp objects, as delay increases complication risks [14]. Contrary to the expected timeline, where abscesses often form 3–4 days post-migration [1], our patient presented after 6 days with only odynophagia. This represents a state of “concealment” with minimal local tissue reaction—a condition we describe as an MIPFB. This atypical presentation likely contributed to the negative initial endoscopy. Furthermore, prior self-administered antibiotics may have suppressed local inflammation, creating false reassurance and compounding the diagnostic difficulty.

Consequently, this case underscores two critical endoscopic strategy shifts. First, one should recognize the esophageal inlet as a high-risk blind spot. Second, in addition to searching for overt ulcers, one should carefully look for subtle mucosal changes such as focal edema. The well-established high success rates of endoscopic removal (>93% in large series) support its primary role [3–6]. Therefore, in selected cases such as this, characterized by unequivocal imaging findings of submucosal embedding and extraesophageal extension, a repeat, meticulous, and targeted endoscopy may be considered a viable and preferable strategy prior to surgical exploration. This approach leverages the efficacy of endoscopy while acknowledging the specific challenges presented by MIPFBs.

## 4 Conclusion

The management of MIPFBs necessitates a paradigm shift from traditional surgical-first approaches towards a prudent, endoscopy-centered strategy. This case underscores that technological advancements in imaging navigation are urgently needed to bridge the current diagnostic–therapeutic gap.

## Data Availability

The original contributions presented in the study are included in the article/Supplementary Material, further inquiries can be directed to the corresponding author.

## References

[1] Wei HX Lv SY Xia B Zhang K Pan CK. Bedside ultrasound-guided water injection assists endoscopically treatment in esophageal perforation caused by foreign bodies: a case report. World J Gastrointest Surg. (2023) 15:1240–6. 10.4240/wjgs.v15.i6.1240 37405102 PMC10315116

[2] Rustemov D Bilal R Tukinov R Nekessov A Dzhenalaev D Ermeshev E, et al. Case report: Unique management strategy for rare case of esophageal foreign body. Front Surg. (2024) 11:1370876. 10.3389/fsurg.2024.1370876 38505410 PMC10948502

[3] He Z Xu Q Fan W Shi J Zhang G Ye F. Non-foreign body-associated risk factors for complications associated with esophageal foreign-body removal and timing of endoscopic treatment: a single-center retrospective study. BMC Gastroenterol. (2024) 24:429. 10.1186/s12876-024-03532-0 39587507 PMC11590538

[4] Li G Wu D Zhou L You D Huang X. Delayed endoscopic management of esophageal sharp-pointed food impaction: an analysis of 829 cases in China. Dig Dis Sci. (2022) 67:3166–76. 10.1007/s10620-021-07133-9 34342753

[5] Sagvand BT Najafali D Yardi I Sahadzic I Afridi L Kohler A, et al. Emergent endoscopy for esophageal foreign body removal: the impact of location. Cureus. (2022) 14:e21929. 10.7759/cureus.21929 35273870 PMC8900722

[6] Topaloglu O Kılıc KN Karapolat S Aydın Y Turkyilmaz A Sengul AT, et al. Diagnosis, treatment, and management of esophageal foreign bodies in patients with mental retardation: a retrospective study from three centers. Turk J Thorac Cardiovasc Surg. (2024a) 32:179–84. 10.5606/tgkdc.dergisi.2024.25724 PMC11197419 38933315

[7] Ribeiro T Mascarenhas Saraiva M Afonso J Brozzi L Macedo G. Predicting factors of clinical outcomes in patients hospitalized after esophageal foreign body or caustic injuries: the experience of a tertiary center. Diagnostics. (2023) 13:3304. 10.3390/diagnostics13213304 37958198 PMC10648504

[8] Yan X Dai G. Esophageal foreign body missed diagnosis; an analysis of 12 cases. Arch Acad Emerg Med. (2023) 11:e65. 10.22037/aaem.v11i1.2102 37840872 PMC10568947

[9] Topaloglu O Buran A Topaloglu ES Karapolat S. A rare reason for intubation: esophageal foreign body lodged in the upper esophageal stricture. Curr Thorac Surg. (2024b) 9:147. 10.26663/cts.2024.027

[10] Rabat SK Sridhar A Makda A Aloysius MM. Fish hook as foreign body: not all foreign bodies can be fished out of the esophagus with endoscopy alone. Cureus. (2022) 14:e28164. 10.7759/cureus.28164. 36158326 PMC9491686

[11] Tuan HX Hung ND Quang NN Tam NT Anh NTH Hoa T, et al. Pulmonary artery penetration due to fish bone ingestion: a rare case report. Radiol Case Rep. (2024) 19:1900–6. 10.1016/j.radcr.2024.02.003 38425774 PMC10904187

[12] Poésy S Sakai O Andreu-Arasa VC. Imaging approach to ingested foreign bodies in the neck. Neuroradiology. (2024) 66:867–81. 10.1007/s00234-024-03348-5 38619570

[13] Chansangrat J. Diagnostic performance of digital radiograph and low-dose computed tomography for the diagnosis of fishbone retention in the oropharynx. Int Arch Otorhinolaryngol. (2022) 26:e401–6. 10.1055/s-0041-1735567 35846813 PMC9282956

[14] Ma Y Tian Y Chen Y Ran H Pan T Xiong X. Combination of gastroscopy and fibro-bronchoscopy facilitates removal of incarcerated fish bone in the esophagus: a case report. Exp Ther Med. (2023) 26:518. 10.3892/etm.2023.12217 37854500 PMC10580255

